# A Multidisciplinary Approach to Implement Personalized Breast Cancer Treatment and Care Plans

**DOI:** 10.3390/curroncol28010075

**Published:** 2021-02-02

**Authors:** Rashida Haq, Amy Kong, Pauline Gulasingam

**Affiliations:** 1 Department of Medicine, Division of Hematology & Oncology, St. Michael’s Hospital, Unity Health, Toronto, ON M5B 1W8, Canada; Amy.Kong@unityhealth.to; 2 Department of Medicine, Faculty of Medicine, University of Toronto, Toronto, ON M5S 1A1, Canada

**Keywords:** breast cancer, survivorship, care plans, health services research, quality improvement

## Abstract

Implementation of survivorship care plans remain a challenge. This quality improvement initiative aims to integrate personalized treatment plans (PTP) and care plans (PCP) into the existing workflow for breast cancer (BC) patients. **Methods:** Phase 1 was to identify multidisciplinary team members to generate and deliver PTP and PCP. Concurrently, Phase 2 was to deliver PTP and PCP to newly diagnosed invasive BC patients at chemotherapy initiation and completion, respectively. Iterative plan, do, study, act (PDSA) cycles were applied to refine the process. The proportion of information completed for PTP and PCP generation and its delivery by the care team were measured. Patient and provider satisfaction were also assessed. **Implementation Process and Results:** The care transfer facilitator (CTF) was identified to complete and deliver PTP, and their data entry increased from 0% to 76%, 80%, 92% consecutively during the last 4 PDSA cycles. PTP and PCP were provided to 85% of eligible BC patients. Patients agreed that PTP helped them to actively participate in their care (88%) and communicate with the oncology care team (86%). Primary care physicians agreed that PTP and PCP had the information needed to “stay in the loop” (80%), and oncologists agreed they should be incorporated into oncology clinics (100%). **Conclusions:** Integrating PTP and PCP generation and delivery into existing workflow has led to an increase in uptake, sustainability and provider buy-in. With limited resources, it remains difficult to find care team members to complete the forms. A dedicated personnel or survivorship clinic is required to successfully implement PTP and PCP as the standard of care.

## 1. Introduction

The most recent Canadian Cancer Society statistics show that, on average, 74 Canadian women will be diagnosed with breast cancer (BC) every day [1]. Due to increasing improvements in screening and treatment, the long-term survival rates after a BC diagnosis are steadily rising. With this process, complexities of cancer care and patient needs have also evolved, emphasizing the need for the development of comprehensive survivorship programs.

Many cancer survivors, including BC patients, continue to report disease-related information needs and would prefer a print format [2]; and they are often dissatisfied with care following cancer treatment [3]. In 2006, the Institute of Medicine (IOM) also issued a milestone report that included 10 recommendations regarding cancer survivorship. The issues receiving the most attention to date have been the provision of a summary of diagnosis, treatment received (treatment summary), future follow-up care plans, and healthy lifestyle recommendations [4].

In a 2009, pilot study conducted at St. Michael’s Hospital (SMH), a multifaceted survivorship care plan (SCP) was developed by clinicians to meet the information and communication needs of BC patients and their primary care physicians. Results of this pilot study suggested that most patients would prefer to receive information at their treatment initiation through the SCPs. They also emphasized the need for more personalized approaches [5]. In response to this and the continued need for comprehensive survivorship care, a standalone database called the care plan application (CP app) was developed in 2015 at SMH. The CP app allowed non-repetitive clinical data to be entered to generate a personalized treatment plan/summary (PTP) and a personalized care plan (PCP), collectively called a personalized, multifaceted care plan (PMCP). In this randomized mixed-methods study patients were stratified into chemotherapy and endocrine therapy. Endocrine patients were given upfront PCP, and patients on chemotherapy were given upfront PTP and PCP at the completion of chemotherapy. The results of this study showed that in BC patients receiving chemotherapy, the PMCP improved self-efficacy scores and empowered BC survivors. Primary care providers found the information to be concise and helpful [6].

Many professional societies, including the American Society of Clinical Oncology [7] and the Commission on Cancer (CoC) [8], have recommended or required the provision of SCPs to cancer patients. One of the strategic objectives of Cancer Care Ontario’s (CCO) Cancer Plan IV (2015–2019) is to ensure that standardized SCPs are developed and communicated to the care team across the cancer care continuum and to facilitate integrated care [9]. Despite the recommendations and reported benefits by patients [10], adoption, implementation and dissemination of SCPs have been slow, low, and sporadic and still remained a challenge [11] across most institutions. The provision of an SCP is not routine practice by the oncologists due to busy clinics and lack of support. Some of the reasons for the lack of widespread adoption are directly related to the implementation process, such as scarcity of resources, lack of designated personnel for SCP preparation and delivery, and limited provider buy-in [12]. Others are related to the conflicting evidence in the literature regarding the benefits of SCPs. To date, despite many studies, including randomized trials, there is no clear evidence that SCPs are effective [13,14,15].

However, many of these studies have primarily assessed the effects of SCPs on distal outcomes, such as patient satisfaction with care [16]. They tend not to address the more proximal, process-related outcomes that we may expect SCPs to influence (e.g., knowledge, communication, and care coordination) or how SCPs are implemented [15]. According to Birken et al. [17], the effectiveness of SCPs needs to be determined, in part, by context and delivery. We need to assess who should develop it, who should deliver it, how should they deliver it, how often, and for whom. Attending to these implementation-related issues would address key barriers to the translation of survivorship research to practice by contributing to implementation research and facilitating the uptake of SCPs [18]. This information will equip us to implement SCPs more effectively and, ultimately, assess their effectiveness in a more rigorous manner [9]. Integrating strategies into the appropriate workflow is critical for successful implementation. The objectives of this quality improvement (QI) project were to implement the PTP and PCP in the oncology clinics at SMH for BC survivors and to analyze the implementation process to identify the barriers and refine it based on an iterative process over time. In addition, patient and provider satisfaction with the PTP and PCP were assessed.

## 2. Materials and Methods

### 2.1. The Intervention

#### 2.1.1. The Care Plan Application (CP App)

The CP app was initially developed for our 2015 study [6]. The CP app allowed non-repetitive clinical data to be entered to generate and save the PTP and PCP electronically. Since then, we have modified and updated the CP app and subsequently tested the web application to ensure complete function for the generation of these documents. The PTP and PCP were designed based on a literature review and reviewing various existing SCP templates, and it contained elements that met current SCP guidelines [6]. This application also has the ability to be modified at minimal cost to generate similar documents for other cancer sites.

#### 2.1.2. Personalized Treatment Plan (PTP)

A paper-based PTP was provided at chemotherapy initiation to newly diagnosed invasive BC patients. This contained patient-specific information, including breast pathology, type of surgery, staging tests, treatment regimen, side effects and information on patients’ care team (Supplementary Materials).

#### 2.1.3. Personalized Care Plan (PCP)

A paper-based PCP was given at chemotherapy completion to the BC survivors who received a PTP. In addition to patient-specific information in the PTP, the PCP included surveillance plans, health promotion information and follow-up care information.

With the recruited patients’ consent, the PTP and PCP were faxed to the primary care physicians. They were also invited to participate in a survey to evaluate the material received.

### 2.2. Implementation Process

**Figure 1:**
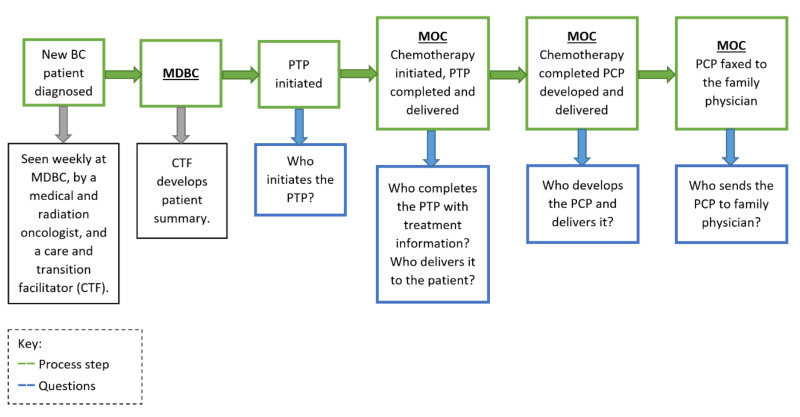
Personalized treatment plan and care plan implementation process. BC = newly diagnosed breast cancer patient, MDBC = multidisciplinary breast clinic, MOC = medical oncology clinic, BC = breast cancer, CTF = care transition facilitator, PTP = personalized treatment plan, PCP = personalized care plan, PCP = primary care providers.

In this QI project (Figure 1), a rapid-cycle improvement methodology using the IOM Model for Improvement framework with iterative PDSA cycles was used to refine the process, and statistical process control charts were used to plot data over time to assess for improvement.

This QI project had two parallel phases: Phase 1was to identify multidisciplinary team members to generate and deliver the PTP and PCP, and secure the standalone CP app to institutional IT server to allow hospital-wide access, and Phase 2 was to deliver PTP and PCP to at least 90% eligible patients (based on the 2015 care plan study data) [6]. The project was conducted over a period of 24-months using PDSA cycles:

Plan: A team was formed, and responsibilities were assigned. Progress meetings were held to review the process and data over time and discuss the ongoing use of the PTP and PCP. Root cause analysis was performed to determine the underlying cause of any barriers to implementation, and the process was reexamined. For example, if we did not meet a target number of eligible patients who received a PTP and PCP, then root cause analysis was initiated to implement changes that addressed the issues. Data over time was studied to assess if the implemented changes resulted in improvement until targets were met.

Do: The action plan was implemented, and data were captured. Any problems, unexpected occurrences, and general observations were documented. A new action plan that implemented a change of ideas to address root causes was implemented.

Study: The implemented plan and outcomes were studied and evaluated. If outcomes were not met, then the process was reexamined to identify areas that could be further improved or whether a new approach was needed (return to the “plan” stage).

Act: Each PDSA cycle took approximately 4–4.5 months to complete. The outcome, process, and balancing measures, as outlined below, were tracked to identify problems with the PTP and PCP implementation and provide information on how and what required improvement. Any changes suggested in the aforementioned PDSA cycle were implemented in the next iteration of the PDSA cycle.

#### 2.2.1. Context of Initiative Implementation

At SMH multidisciplinary breast clinic (MDBC), new BC patients are seen weekly by a medical oncologist, radiation oncologist, and care transition facilitator (CTF, a registered nurse) to discuss management. Prior to this initiative, the CTF routinely created a paper-based patient summary in isolation that was then shared with the oncologists before each clinic. Patients needing follow-up and receiving any type of treatment (e.g., chemotherapy) are transferred from MDBC to medical oncology clinic (MOC) subsequently. During the transfer of care, the CTF shared multiple word documents with the MOC staff.

During the first two QI cycles, all steps related to PTP and PCP development and delivery were conducted by the research staff. Developing each individual document was a time-consuming process. Hence, during the third PDSA cycle, we identified that the integration of work related to the PTP and PCP development into the oncology care workflow was imperative to overcome the many barriers associated with their successful implementation. During subsequent QI meetings, the potential involvement of care team members whose role would fit with the development of the PTP and PCP was explored. The CTF was then identified as the most suitable care team member to initiate the entry of patient information into the CP app. However, to efficiently incorporate the use of the CP app into their workflow, it had to be modified to generate a document useful to the CTF. This resulted in the development of the transfer of accountability (TOA) document.

Transfer of accountability document (TOA): This was developed through the CP app to replace the patient summary and multiple other paper-based patient information documents sent from MDBC to MOC during care transfer. The TOA allowed patient information to be shared electronically with MDBC and MOC teams, and the use of one platform (the CP app) avoided duplication of documentation. The TOA also acted as a paper-based follow-up plan given to MDBC patients.

As the CP app was a standalone application, the possibility to migrate it to a secure institutional server was explored in parallel to PCP and PTP implementation in efforts to increase user access to PTP and PCP.

#### 2.2.2. Primary Outcome Measure

The proportion of eligible patients who received a PTP and PCP (target was to deliver PTP and PCP to 90% of eligible patients). The investigators arrived at this percentage based on the 2006 IOM report recommends that every cancer patient receives an SCP [4].

#### 2.2.3. Primary Process Measure

The proportion of information completed by the patient’s care team to generate the PTP.

#### 2.2.4. Balancing Measures

Patient satisfaction with the PTP and PCP was assessed mid- and end-of-chemotherapy and 3 months post-chemotherapy using a modified patient CARE-path questionnaire [19]. Similarly, provider satisfaction was assessed using a modified provider CARE-path questionnaire [19]. Provider satisfaction was evaluated once to primary care physicians at their patient’s chemotherapy completion and to oncologists around 6 months after starting the project. Both patient and provider questionnaires used a Likert scale with five responses: strongly disagree (1), disagree (2), undecided (3), agree (4), and strongly agree (5).

Patients were further assessed using a communication and attitudinal self-efficacy scale for cancer (CASE-cancer) [20], which was administered at chemotherapy initiation and completion.

### 2.3. Participants

Eligible participants were newly diagnosed invasive BC patients seen at SMH MDBC or MOC expected to receive neo/adjuvant chemotherapy, cancer care team members (oncologists and care transition facilitator) and primary care physicians of the patients who received PTPs and PCPs. We included this patient population based on our 2015 study findings [6].

#### 2.3.1. Patient Recruitment

A sample size of 120 participants with an estimated 90% of eligible subjects to receive the PTP and PCP gave us a 95% confidence interval of 0.852, 0.933. To account for dropout, this sample size was inflated by 10%.

#### 2.3.2. Statistical Analysis

The primary outcome was reported as the proportion of eligible subjects that received a PTP and PCP with 95% confidence intervals. All outcomes were summarized descriptively by timepoint and overall. Continuous outcomes were summarized with median, interquartile ranges, as well as frequency and percent. A mixed-effects model was used to examine whether the CARE-path score changed over time. Values were not adjusted for baseline values as there were no baseline values. Categorical outcomes will be summarized with counts and proportions.

## 3. Results

Institutional REB approval was obtained in March 2017.

We conducted 7 PDSA cycles as follows (Table 1):

- PDSA 1: April–July 2017
- PDSA 2: August–November 2017
- PDSA 3: December 2017–March 2018
- PDSA 4: April–July 2018
- PDSA 5: August–November 2018
- PDSA 6: December 2018–March 2019
- PDSA 7: April–July 2019

**Figure 2:**
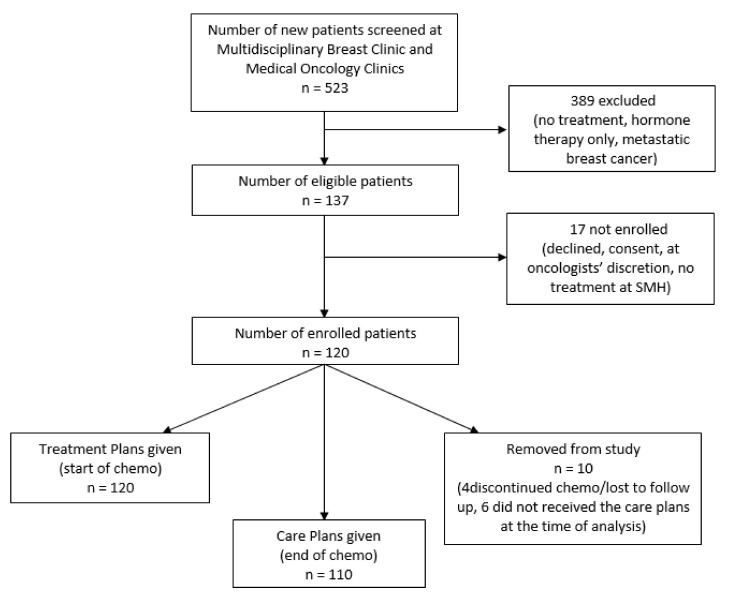
Patient recruitment flow chart. MDBC = multidisciplinary breast clinic, MOC = medical oncology clinic, BC = breast cancer, PTP = personalized treatment plan, PCP = personalized care plan.

Among eligible BC patients, 120 were enrolled, with 110 who received both the PTPs and PCPs (Figure 2). The mean age of this group was 53 years (range 24 to 78 years). Diagnoses included 101 invasive ductal carcinomas (91.8%), 5 invasive lobular carcinomas (4.5%), and 4 others (3.6%). Out of the 110 patients, 70 (63.5%) and 40 (36.4%) received adjuvant and neoadjuvant chemotherapy, respectively.

### 3.1. Primary Outcome Measure Results

During the 7 PDSA cycles, 88%, 87%, 92%, 87%, 83%, 87% and 88% of eligible BC patients were provided with PTPs and PCPs, respectively. Overall, 87% of eligible patients have received PTPs and PCPs.

### 3.2. Process Measure Results

**Figure 3:**
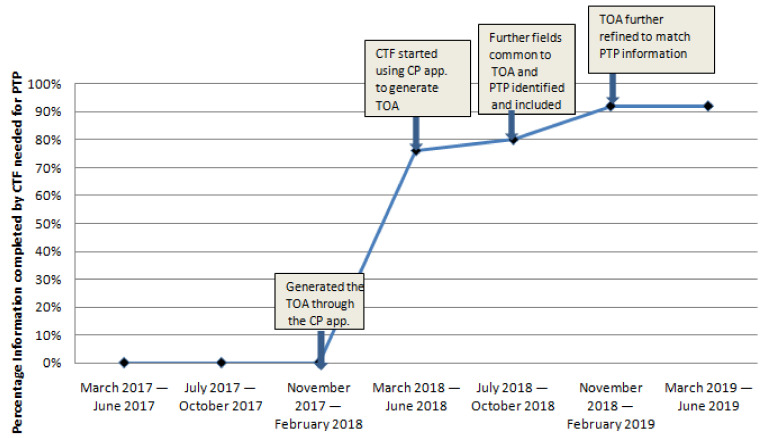
Percentage of information completed by care transfer facilitator forgenerating the PCP. TOA = transfer of accountability document, CTF = care transfer facilitator

During the initial PDSA cycles, all information needed to generate the PTP and PCP was entered by the research staff. With the development of the TOA, an increasing amount of data common to TOA and PTP were identified and TOA was updated through the CP app. This increased the data entry needed to generate the PTP and PCP by CTF from 0% to 76%, 80%, 92% consecutively during the next 4 PDSA cycles (Figure 3). After TOA was developed, CTF generated and shared a TOA document with the care team for every new BC patient that attended the MDBC (100%).

### 3.3. Balancing Measure Results

Patient CARE-path questionnaire: Patient satisfaction with the PTP and PCP is shown in Table 2 captured at mid- and end-of-chemotherapy and 3-months post-chemotherapy. Quantitative values can be found in Appendix A, where n refers to the total number of observations available and does not reflect missing data. The responses for each component had been summarized using median and interquartile range (IQR) at the 3 data points.

Patient CARE-path questionnaire responses were consistent overall time points with high levels of agreement. In particular, at least 75% of patients agreed that the PTP helped with communication with their primary care physician, overall communication and answered their care-related questions.

A linear mixed-effects model examined the change in the CARE-path over time after adjustment for age and chemotherapy type and found no statistically significant change in score over time.

Provider satisfaction questionnaire: Results include feedback from 4 oncologists and 24 (out of 85 contacted) primary care providers, as shown in Table 3. For the providers where there was only one time point, there were 24 providers that had less than half of the responses missing and were included. Table 3 shows the breakdown of the score on each component given the percentage.

Providers were generally favorable of the PTP and PCP implementation with an average score of 3.8 (SD = 0.8) out of 5, and 23 out of 25 of them were neutral or disagreed that it was time-consuming.

CASE-cancer scale: CASE-cancer score after adjustment for age and chemotherapy type, found no statistically significant change in score over time.

### 3.4. Collaboration with Institutional IT Department

We were able to collaborate with the institutional IT department to maintain the app for sustainability. The IT department also supported the migration of the app to a secure hospital server and making the app available as a computer desktop icon that can be logged in by staff using their institutional log-in information. With the migration and installation as a desktop icon, the CP app and documents it generated (TOA, PTP, and PCP) have become directly accessible to SMH users. Brief training sessions for the users of the CP app were also conducted.

## 4. Discussion

Overall, during this project, we were able to provide the PTP and PCP to 87% of eligible BC patients, which was closer to our initial target of 90%. This was the first time a cancer survivor of any site received the PTP and PCP at our institution, in accordance with the current Ontario provincial guidelines [9]. In addition, we primarily assessed the process related outcomes and proximal patient-related outcomes, which were favorable.

Barriers to developing PTPs and PCPs were eliminated as a result of developing the CP app to generate multiple clinical documents through a non-repetitive database. This included the TOA, which was created specifically to replace a patient summary used in the standard of care and seamlessly integrate the use of the CP app into the workflow of the care transfer facilitator (CTF)The CTF became the initial care team member to enter clinical data into the CP app, providing shared information that is found in the PTP and PCP. This minimized the amount of data entry needed to complete the PTP and PCP. The remaining information required for completion of the PTP included chemotherapy regimen and start date, and investigation results, and for PCP, alterations to the chemotherapy regimen, side effects encountered and follow-up. With time, the CTF was involved in the completion and delivery of the PTP.

The CTFs and MDBC team conducted a project on the implementation of the TOA and patient follow-up plans through the Best Practice Spotlight Organization in 2018 [21]. Results from the care team survey (n = 13) showed: 100% of the staff agreed the TOA was helpful in the patient’s care transition, and 82% agreed the CP app is easy to navigate. Patients’ qualitative feedback highlighted the informativeness of having a paper-based TOA as a follow-up plan.

Furthermore, the use of the TOA allowed patient information to be shared electronically while eliminating the use of paper-based documents. The use of this single common platform for care team members also streamlined the care process and facilitated better care coordination between the MDBC and MOC teams, particularly during the transfer of care. In addition, the migration to a password protected secure hospital server and making it available as a desktop icon has increased the use of the CP app and its documents (TOA, PTP and PCP) by the patient care team.

The CARE-path satisfaction questionnaire results showed that there were high patient and provider satisfaction with PTP and PCP and the overall positive trend towards the implementation of PTPs and PCPs. Patient responses were consistent over all time points with high levels of agreement. At mid- and end-of-chemotherapy, cumulative results of 4 (agree) and 5 (strongly agree) out of five responses showed that: patients agreed that PTP helped with communicating with the oncology care team (86%) and it helped to actively participate in their care (88%). Furthermore, primary care physicians agreed that PTP and PCP had the information needed to “stay in the loop” (80%) and oncologists agreed that PTP and PCP should be incorporated into the MOC (100%). The ability of the CP app to generate tailored, brief and patient-centered PTP and PCP may be the reason for the high patient and provider satisfaction.

Identifying and involving clinical staff in the generation and delivery of SCPs was one of the greatest barriers to implementation [22]. Some cancer programs have hired support staff to ensure that SCPs are developed and delivered to survivors and their follow-up care providers, with the goal of facilitating sustained communication and coordination between oncology care providers and follow-up care providers, meeting the letter of the standard, but not the intent [14]. A 2013 study has indicated that oncology nurses and nurse practitioners are well-positioned to create and deliver SCPs, transitioning patients from oncology care to a PCP [23]. Nicolaije et al. [24] noted that “oncology providers in the SCP care hospitals were free to choose” who delivered SCPs to promote fit with their clinical practice. We believe this ability to adapt the intervention (in our case, the CP app) is crucial for promoting SCP implementation. In this project, the PTP and PCP were implemented by integrating the data capture for multiple benefits within the existing workflow and involvement of existing members of the care team (i.e., the CTF). This has led to increased favorability of care plan implementation, communication, care coordination and provider buy-in.

In developing a survivorship care plan, Ganz said there is some uncertainty about when the process should start [25]. For patients with BC, some clinicians are comfortable preparing a care plan when women have finished their primary treatment and are starting endocrine therapy [26], as stated by the IOM recommendations [4]. However, during our 2013 study on designing a multifaceted survivorship care plan study, it was shown that patients did not focus exclusively on the posttreatment period but instead spoke of evolving needs throughout their cancer journey and the need for care planning from the time of diagnosis [5]. In evaluating their original end-of-treatment approach, the United Kingdom National Cancer Survivorship Initiative (NCSI) has realized that many of the issues that arose at that time could have been handled earlier in the treatment trajectory [27]. They have recommended expanding the understanding of survivorship to cover the entire cancer care continuum reflecting that people want and need information, individualized support, and care planning from diagnosis onward, not just after treatment completion. Similarly, we recommend creating the treatment plan at treatment initiation, updated as necessary throughout the cancer care trajectory, and delivering the care plan at treatment completion. This is to empower BC patients to be able to care for themselves as in the chronic diseases model.

Through this QI project, we were able to develop and deliver the PTP without the involvement of the research staff; however, we were not able to update and deliver the PCP in a similar manner at the completion of chemotherapy.

During the implementation of PTP in later PDSA cycles, more responsibility fell on the CTF. However, as noted in the literature, having one SCP provider, which is what most institutions have, who is expected to be the one to compile data into the care plan, present the document to the patient and educate them is unsustainable [28]. Multiple efforts were made to involve MOC team members (medical oncologists and pharmacists) in the completion (inputting chemotherapy regimen and start date) and delivery of PTPs. Although they were supportive of PTP and PCP implementation, they did not get involved in the development of it. The CP app, even after transferring to a hospital server, still functioned independently of the hospital electronic health record platform, which all providers used in the MOC. Having a separate computer interface (CP app) for PTP and PCP generation may have been a deterrent to the development and use of care plans by the oncology team.

Furthermore, lack of specific training, no reimbursement, and time constraints still posed barriers to PTP and PCP implementation. Increasing the use of PTPs and PCPs may be facilitated by templates that capture automated data from the electronic health record [28]. Lack of institutional buy-in due to limited resources also has hindered the implementation of SCPs. There is a need to foster active leadership to make SCP implementation a priority.

In the United States, the majority began using SCPs because of professional societies’ recommendations [7,8]. Other than the recommendations, there should be a mechanism to monitor the providers’ compliance rate in issuing SCPs. Shulman [29] has mentioned that some have recommended that the CoC eliminate the numerical targets and focus on the components of a hospital’s survivorship program. Others have said that the numerical targets push programs to invest more seriously in survivorship [30]. As stated above, it was more challenging to implement the PCPs than the PTPs, as there was no process to update the necessary information and deliver at the end of active treatment. Furthermore, as in many programs, PCP/SCP was the sole element of our survivorship program with no posttreatment meeting. This is unlikely to be effective as there are no mechanisms in place to implement the plan’s recommendations [31]. Therefore, we believe that for successful PCP/SCP implementation, it needs to be woven into a more comprehensive cancer survivorship clinic following the end of active treatment [31].

Further research about the effectiveness of care plans is also needed to be conducted, choosing the most relevant outcome measures.

## 5. Conclusions

This QI initiative aligns with the CCO’s strategic objective of providing treatment plans and care plans to cancer patients. Implementation of PTP and PCP by integrating them within the existing workflow and involvement of the multidisciplinary care team has led to an increase in their uptake, better care coordination, and provider buy-in. There is high patient and provider satisfaction with PTP and PCP. Currently, we are in the process of integrating PTP for BC patients as a standard of care. The oncology team is interested in delivering the PTP and PCP to patients yet not willing to completing the forms in the busy clinics. It is evident that dedicated personnel or a survivorship clinic is required to implement PCP and PTP as a standard of care.

## Figures and Tables

**Table 1:** Plan, do, study, act (PDSA) cycles and project progress.

Each PDSA cycle started with: QI committee meeting Patient enrollment Eligible breast cancer patients receiving a PTP and PCP	
PDSA 1 Apr–Jul 2017	All activities related to PTP and PCP preparation and delivery conducted by research staff. Involvement of oncology care team members in PTP and PCP generation, preparation and delivery explored.
PDSA 2 Aug–Nov 2017	All activities related to PTP and PCP preparation and delivery conducted by research staff. Explored CP app capability to generate existing oncology care documents used by care team members.
PDSA 3 Dec 2017–Mar 2018	TOA document created and integrated in the CP app to replace patient summary used by CTF. CTF of multidisciplinary breast clinic identified as the care team member to start creating the PTP. Standalone CP app migrated to SMH secure server.
PDSA 4 Apr–Jul 2018	CTF started using the CP app to generate TOAs. With the shared information, PTP and PCP initiated. CP app made available as a SMH desktop shortcut for care team members.
PDSA 5 Aug–Nov 2018	As TOA was further updated, more fields needed for PTP and PCP got completed. Explored the ability involve the oncologists and pharmacists to complete the PTP.
PDSA 6 Dec 2018–Mar 2019	In consultation with clinic managers and CTF, the CTF was identified as the team member to complete and deliver PTP.
PDSA 7 Apr–Jul 2019	CTF took up the responsibility to generate and deliver the PTP at medical oncology clinic.

PTP = personalized treatment plan, PCP = personalized care plan, CP app = care plan application, CT = care and transition facilitator, TOA = transfer of accountability, SMH = St. Michael’s Hospital.

**Table 2:** Level of agreement reported by patients regarding satisfaction with the personalized treatment and care plan.

	Mid Chemotherapy	End of Chemotherapy	3-Months Post Chemotherapy	*p*-Value
**Number of patients who completed the form**	109	113	81	NA
**Prepared to live as cancer survivor (median (IQR))**	4.00 (3.00, 4.00)	4.00 (3.00, 4.07)	4.00 (3.00, 4.00)	0.55
**Better communicate with medical team** **(median (IQR))**	4.00 (4.00, 4.00)	4.00 (4.00, 5.00)	4.00 (4.00, 5.00)	0.13
**More easily communicate with other health care providers (median (IQR))**	4.00 (4.00, 5.00)	4.00 (4.00, 5.00)	4.00 (4.00, 5.00)	0.72
**Actively participate in medical management of cancer** **(median (IQR))**	4.00 (4.00, 4.00)	4.00 (3.50, 4.00)	4.00 (3.00, 4.47)	0.18
**Enlist family’s support (median (IQR))**	4.00 (4.00, 5.00)	4.00 (4.00, 5.00)	4.00 (3.00, 4.00)	0.06
**Deal more effectively with side effects of treatment** **(median (IQR))**	4.00 (3.00, 4.00)	4.00 (4.00, 5.00)	4.00 (3.00, 4.00)	0.23
**Deal more effectively with pain (median (IQR))**	4.00 (3.00, 4.00)	4.00 (3.00, 4.00)	3.00 (3.00, 4.00)	0.34
**Reduce risk of delayed complications for treatment** **(median (IQR))**	4.00 (3.00, 4.00)	4.00 (3.00, 4.00)	4.00 (3.00, 4.00)	0.18
**Better understand lifestyle factors leading to cancer onset (median (IQR))**	4.00 (3.00, 4.00)	4.00 (3.00, 4.00)	4.00 (3.00, 4.00)	0.12
**Better understand lifestyle changes that reduce recurrence risk (IQR))**	4.00 (3.00, 4.00)	4.00 (3.00, 4.00)	4.00 (3.00, 4.00)	0.61
**Make nutritional changes which aid recovery (IQR))**	4.00 (3.00, 4.00)	4.00 (3.00, 4.00)	4.00 (3.00, 4.00)	0.51
**Make physical activity changes to aid recovery (median (IQR))**	4.00 (3.00, 4.00)	4.00 (3.00, 4.00)	4.00 (3.00, 4.00)	0.81
**Provided helpful advice on issues related to return to work (median (IQR))**	3.44 (3.00, 4.00)	3.67 (3.00, 4.00)	3.00 (3.00, 4.00)	0.77
**Be informed of community resources available (median (IQR))**	4.00 (3.00, 4.00)	4.00 (3.00, 4.00)	4.00 (3.00, 4.00)	0.39
**More easily communicate with other health care providers (median (IQR))**	4.00 (3.53, 4.00)	4.00 (4.00, 5.00)	4.00 (3.00, 4.00)	1

Patient satisfaction with the PTP = personalized treatment plan and PCP = personalized care plan were collected at mid- and end-of-chemotherapy, and 3-months post-chemotherapy using a questionnaire with a Likert scale with five responses: strongly disagree (1), disagree (2), undecided (3), agree (4), and strongly agree (5). The table shows the responses for each component summarized using median and interquartile range (IQR) at the 3 data points.

**Table 3:** Responses were reported by primary care providers regarding satisfaction with the personalized treatment and care plan and how it has helped their patients with the following.

	1	2	3	4	5
**Prepared to live their lives as cancer survivor (%)**	4	0.0	20.0	28.0	48.0
**Better communicate with their medical team (%)**	4.2	0.0	12.5	16.7	66.7
**Reduce risk of delayed complications for treatment (%)**	0.0	4.2	41.7	8.3	45.8
**Better deal with depression or depressed mood (%)**	4.2	0.0	50.0	20.8	25.0
**PTP and PCP helped to improve quality of patient care (%)**	4.0	0.0	16.0	16.0	64.0
**PTP and PCP should be incorporated into oncology clinic (%)**	4.2	0.0	4.2	16.7	75.0
**PTP and PCP has all the information needed (%)**	4.2	4.2	0.0	25.0	66.7
**Better understand lifestyle changes that reduce recurrence risk (%)**	4.5	9.1	22.7	22.7	40.9
**Better understand lifestyle factors leading to cancer onset (%)**	8.3	4.2	25.0	16.7	45.8
**Actively participate in medical management of cancer (%)**	4.2	0.0	20.8	37.5	37.5
**Make nutritional changes which aid recovery (%)**	4.2	0.0	20.8	37.5	37.5
**Deal more effectively with pain (%)**	0.0	0.0	33.3	29.2	37.5
**Make physical activity changes to aid recovery (%)**	8.3	0.0	29.2	37.5	25.0
**Ask right questions about treatment (%)**	4.2	4.2	12.5	33.3	45.8
**Be informed of community resources available (%)**	8.3	00.0	25.0	25.0)	41.7
**Deal more effectively with side effects of treatment (%)**	4.2	00.0	20.8	25.0	50.0
**Better deal with stressful emotions and anxiety (%)**	4.2	00.0	33.3	33.3	29.2
**PTP and PCP is more time-consuming to use (%)**	36.0	16.0	40.0	4.0	4.0

Provider satisfaction with the PTP = personalized treatment plan, and PCP = personalized care plan were collected at one time point using a questionnaire with a Likert scale with five responses: strongly disagree (1), disagree (2), undecided (3), agree (4), and strongly agree (5). The table shows the breakdown of the score on each component using percentages.

## Data Availability

Not Applicable.

## Supplementary Materials

The following are available online at https://www.mdpi.com/1718-7729/28/1/75/s1.

## Appendix A

**Table A1:** Responses reported by patients regarding satisfaction with the personalized treatment and care plan.

	Mid-Chemotherapy						End of Chemotherapy						3-Months Post Chemotherapy					
	n	1	2	3	4	5	n	1	2	3	4	5	n	1	2	3	4	5
**Prepared to live as cancer survivor**	109	7 (6.5)	6 (5.6)	28 (26.2)	45 (42.1)	21 (19.6)	117	2 (1.8)	5 (4.5)	27 (24.5)	49 (44.5)	27 (24.5)	85	3 (3.8)	3 (3.8)	27 (33.8)	32 (40.0)	15 (18.8)
**More easily communicate with other health care providers**	109	4 (3.7)	5 (4.6)	18 (16.7)	58 (53.7)	23 (21.3)	117	1 (0.9)	1 (0.9)	18 (17.0)	56 (52.8)	30 (28.3)	85	1 (1.2)	3 (3.8)	19 (23.8)	38 (47.5)	19 (23.8)
**Better communicate with medical team**	109	2 (1.8)	4 (3.7)	13 (11.9)	60 (55.0)	30 (27.5)	117	0 (0.0)	3 (2.7)	15 (13.3)	55 (48.7)	40 (35.4)	85	0 (0.0)	1 (1.2)	17 (21.0)	38 (46.9)	25 (30.9)
**Reduce risk of delayed complications for treatment**	109	2 (1.9)	7 (6.7)	34 (32.4)	40 (38.1)	22 (21.0)	117	0 (0.0)	2 (1.8)	39 (35.8)	43 (39.4)	25 (22.9)	85	1 (1.3)	2 (2.5)	34 (43.0)	30 (38.0)	12 (15.2)
**Better deal with depression or depressed mood**	109	1 (0.9)	7 (6.5)	44 (41.1)	42 (39.3)	13 (12.1)	117	2 (1.9)	3 (2.8)	40 (37.0)	46 (42.6)	17 (15.7)	85	0 (0.0)	7 (8.6)	37 (45.7)	28 (34.6)	9 (11.1)
**Enlist family’s support**	109	1 (0.9)	5 (4.6)	19 (17.4)	54 (49.5)	30 (27.5)	117	0 (0.0)	3 (2.7)	24 (21.6)	51 (45.9)	33 (29.7)	85	1 (1.2)	1 (1.2)	24 (29.6)	38 (46.9)	17 (21.0)
**Better understand lifestyle changes that reduce recurrence risk**	109	6 (5.6)	10 (9.3)	30 (28.0)	42 (39.3)	19 (17.8)	117	3 (2.7)	4 (3.6)	23 (20.9)	56 (50.9)	24 (21.8)	85	0 (0.0)	7 (8.6)	25 (30.9)	37 (45.7)	12 (14.8)
**Better understand lifestyle factors leading to cancer onset**	109	7 (6.5)	7 (6.5)	34 (31.5)	45 (41.7)	15 (13.9)	117	3 (2.8)	7 (6.4)	28 (25.7)	48 (44.0)	23 (21.1)	85	0 (0.0)	6 (7.4)	23 (28.4)	40 (49.4)	12 (14.8)
**Actively participate in medical management of cancer**	109	1 (0.9)	1 (0.9)	19 (17.4)	62 (56.9)	26 (23.9)	117	2 (1.9)	1 (0.9)	26 (24.1)	57 (52.8)	22 (20.4)	85	0 (0.0)	2 (2.5)	20 (25.0)	38 (47.5)	20 (25.0)
**Make nutritional changes which aid recovery**	109	4 (3.7)	8 (7.4)	23 (21.3)	52 (48.1)	21 (19.4)	117	2 (1.8)	1 (0.9)	31 (28.2)	51 (46.4)	25 (22.7)	85	0 (0.0)	4 (4.9)	26 (32.1)	39 (48.1)	12 (14.8)
**Deal more effectively with pain**	109	2 (1.9)	7 (6.5)	36 (33.6)	45 (42.1)	17 (15.9)	117	0 (0.0)	7 (6.4)	32 (29.4)	44 (40.4)	26 (23.9)	85	0 (0.0)	4 (4.9)	39 (48.1)	26 (32.1)	12 (14.8)
**Make physical activity changes to aid recovery**	109	5 (4.7)	4 (3.7)	27 (25.2)	52 (48.6)	19 (17.8)	117	2 (1.9)	3 (2.8)	32 (29.6)	53 (49.1)	18 (16.7)	85	0 (0.0)	1 (1.2)	28 (34.6)	38 (46.9)	14 (17.3)
**Ask right questions about treatment**	109	1 (0.9)	3 (2.8)	8 (7.3)	71 (65.1)	26 (23.9)	117	0 (0.0)	1 (0.9)	19 (16.8)	59 (52.2)	34 (30.1)	85	0 (0.0)	2 (2.5)	14 (17.3)	43 (53.1)	22 (27.2)
**Be informed of community resources available**	109	6 (5.6)	3 (2.8)	26 (24.1)	53 (49.1)	20 (18.5)	117	0 (0.0)	5 (4.4)	26 (23.0)	61 (54.0)	21 (18.6)	85	2 (2.5)	4 (4.9)	24 (29.6)	37 (45.7)	14 (17.3)
**Provided helpful advice on issues related to return to work**	109	10 (11.2)	10 (11.2)	30 (33.7)	27 (30.3)	12 (13.5)	117	6 (6.4)	5 (5.3)	40 (42.6)	30 (31.9)	13 (13.8)	85	4 (6.2)	5 (7.7)	32 (49.2)	19 (29.2)	5 (7.7)
**Deal more effectively with side effects of treatment**	109	1 (0.9)	7 (6.4)	25 (22.9)	51 (46.8)	25 (22.9)	117	0 (0.0)	4 (3.5)	23 (20.4)	54 (47.8)	32 (28.3)	85	0 (0.0)	2 (2.5)	27 (33.3)	37 (45.7)	15 (18.5)
**Better deal with stressful emotions and anxiety**	109	2 (1.8)	8 (7.3)	36 (33.0)	47 (43.1)	16 (14.7)	117	1 (0.9)	2 (1.8)	30 (26.5)	56 (49.6)	24 (21.2	85	0 (0.0)	6 (7.4)	31 (38.3)	34 (42.0)	10 (12.3)
