# Supplementary material for: A Multidisciplinary Approach to Implement Personalized Breast Cancer Treatment and Care Plans
Source: Curr Oncol. 2021 Feb 2;28(1):767–82. doi: 10.3390/curroncol28010075 (PMC7985785; doi:10.3390/curroncol28010075)
Supplement: Supplementary file 1 [file curroncol-28-00075-s001.pdf]

St. Michael's
Inspired Care.
Inspiring Science.

My Oncology Treatment Plan (Sample)

7 3 8 4 3

MRN:	12152016
Name:	Jane Smith
Date of Birth:	December 14, 1950 (69 years old)
Other Medical Conditions:	Hypertension, dyslipidemia, type 2 diabetes, and anxiety
Current Medications:	Valsartan, Metformin, Atorvastatin, Ativan
Allergies:	None
Family History:	Second degree relative - Breast cancer
Previous Breast Cancer:	No

Surgical Information

Dates:

Date of Biopsy:	11th of Aug, 2016
Date of Surgery:	1st of Sep, 2016
Type of Surgery:	Right Breast Mastectomy / Immediate Reconstruction

Pain, swelling and numbness at the site of surgery and radiation are common and will resolve over time. Follow recommended exercises to improve arm mobility.

Pathology:

Diagnosis:	Right Invasive Ductal Carcinoma
Size:	1.5 cm
Grade:	III
Margins:	negative (closest is 5 mm, anterior), lymphovascular invasion present
Sentinel Nodes:	4 nodes negative and 2 nodes positive for malignancy (2/6)
Hormone Receptors:	ER positive (35%), PR positive (5%), Her2neu positive
Stage:	IV : (T1N0(i+)M1)
Pretreatment Echocardiogram/MUGA:	EF 65%
Bone Mineral Density:	Normal (6th of Jun, 2018)
Height:	157.00 cm
Pretreatment weight:	49.00 kg
Pretreatment BSA:	1.46 m^2
Pretreatment BMI:	19.9

Staging Tests:

Chest X-ray:	*Follow-up required* (15th of Jun, 2016)<br>- Nodule seen in left lung, in accordance with previous COPD.
Ultrasound of Abdomen & Pelvis:	*Pending* (27th of Apr, 2016)
CT of Thorax:	*Normal* (8th of Jul, 2016)
Bone Scan:	*Abnormal* (22nd of Sep, 2016)<br>- Nodule detected in right hip region.

Venous Access: Portacath inserted on 22nd of Jul, 2019

Oncotype Test

N/A

EndoPredict Test

EPclin Risk Score: 2.6 (low risk)
10-Year Likelihood of Distant Recurrence: 5% with 5 years of endocrine therapy alone

Genetic Screening

Genetic test has been requested, results pending.

Drug Coverage

Patient has private drug coverage.

Psychosocial Support

Patient requires additional support from social worker

Adjuvant Chemotherapy

You have made the decision with your medical oncologist to undergo chemotherapy to reduce the risk of recurrence and improve survival. Every patient's experience with treatment is different and all measures will be taken to prevent or reduce the side effects.

Regimen: AC (Doxorubicin, Cyclophosphamide) (Dose Dense) + Paclitaxel (Biweekly) + Trastuzumab

AC every 2 weeks for 4 cycles, followed by Paclitaxel and Trastuzumab every 2 weeks for 4 cycles, followed by Trastuzumab alone every 3 weeks for 18 cycles.

Treatment dates*:

2nd of Jan, 2019	7th of Aug, 2019
16th of Jan, 2019	28th of Aug, 2019
30th of Jan, 2019	18th of Sep, 2019
13th of Feb, 2019	9th of Oct, 2019
27th of Feb, 2019	30th of Oct, 2019
13th of Mar, 2019	20th of Nov, 2019
27th of Mar, 2019	11th of Dec, 2019
10th of Apr, 2019	1st of Jan, 2020
24th of Apr, 2019	22nd of Jan, 2020
15th of May, 2019	12th of Feb, 2020
5th of Jun, 2019	4th of Mar, 2020
26th of Jun, 2019	25th of Mar, 2020
17th of Jul, 2019	15th of Apr, 2020

Your last chemotherapy treatment is on the underlined date, 10th of Apr, 2019. Your Trastuzumab treatment will continue afterwards on the listed dates.

* Tentative dates; may change according to varying circumstances

Possible Early Side Effects of Chemotherapy:

Allergic reactions

Fatigue
Fever
Hair loss
Joint aches and pains
Nausea and vomiting
Neuropathy (tingling and numbness)
Skin changes
Sores in the mouth
Diarrhea.

Possible Late Side Effects of Chemotherapy:
Fatigue
Heart problems
Joint aches and pains
Reproductive changes.

Discuss any side effects with your oncology team or consult study website.

Medications You May Be Given To Prevent Side Effects

- Ondansetron - To prevent nausea and vomiting.
- Dexamethasone - To reduce nausea and to prevent allergic reaction with docetaxel.
- Aprepitant - To treat nausea.
- Prochlorperazine - To treat nausea.
- Senokot - To prevent and/or treat constipation.
- Nystatin - To treat mouth sores and fungal infection.
- Grastofil - To stimulate the growth of white blood cells, which help your body fight infections.
- Loperamide - To treat diarrhea.
- Tylenol - For pain

Endocrine or Hormone Therapy (4 weeks after completion of Chemotherapy)

Endocrine or hormone therapy will be prescribed by the oncologist in addition to chemotherapy for your hormone-receptor-positive breast cancer to further reduce risk of recurrence.
Regimen: Tamoxifen

Possible Common Side Effects: Hot flashes, vaginal dryness, memory loss, mood changes, fatigue/weakness, mild nausea/vomiting, and dry, itchy skin (rash).

Possible Less Common Side Effects: Mild swelling in arms and legs (puffiness), blood clots, unusual vaginal bleeding, and endometrial cancer (rare).

For details of any side effects consult your Oncology team and the study website.

Radiation Therapy (4 weeks after completion of Chemotherapy)

A referral has been sent to Princess Margaret Hospital for discussion on your radiation therapy.

Since radiotherapy is localized to a single area (as opposed to chemotherapy, which affects the whole body), skin side effects are mainly found where the radiation is given.
Possible Side Effects of Radiation: The affected area may look like a sunburn, being

pink/red, sore, and itching.

Manage skin side effects:

- Wear loose-fitting clothes so that there is less irritation to the affected area.
- If your doctor has recommended skin salves and ointment, use them as instructed.
- Before your treatment begins, moisturize the skin area that is going to receive radiation.
- Moisturize frequently.

For more information about radiation therapy check www.UHNPatientEducation.ca.

Preparing For Treatment

- Take medications recommended to reduce and help manage side effects as directed.
- Make a list of questions that you may have for your medical team.
- If possible, bring a family member or friend to the clinic visits.
- Maintain a healthy diet and on days of treatment, eat a light breakfast.
- Bring any medications, *supplements*, minerals, herbal or *alternative medicines* that you are using to the Medical Day Care on the day of your treatment so your medical team can make sure they don't conflict with your chemotherapy treatment.
- Carry out gentle exercises (such as walking, yoga, or tai chi) and/or join exercise groups (such as the Toronto Rehab Breast Rehab Program).
- Ask for a referral to a social worker for support if necessary.

Care Team Information

Surgeon
Oncologist
Radiation Oncologist
Primary Care Physician
Care Transfer Facilitator
Social Worker
Medical Day Care
Dietician
Chaplain

This treatment plan is provided for your information and education about diagnosis and plan of treatment and management of side effects. Share this with your Primary Care Physician. This treatment plan does not replace the advice of your Oncology Care team. Hope this information for support will help you during your treatment, however your oncology team can be contacted with any further questions or concerns.

Please visit breastcancer09.utorontoeit.com/Home.html for further information on side effects, healthy lifestyle, and support services during your diagnosis, treatment and follow-up.

A care plan will be given to you upon completion of your chemotherapy.

The drug information provided with this document was obtained through the Drug Formulary information resource on the Cancer Care Ontario (CCO) website. Please be advised of CCO’s disclaimer included with this document.
